# Association of *TLR2* and *TLR4* Polymorphisms with Risk of Cancer: A Meta-Analysis

**DOI:** 10.1371/journal.pone.0082858

**Published:** 2013-12-20

**Authors:** Longbiao Zhu, Hua Yuan, Tao Jiang, Ruixia Wang, Hongxia Ma, Shuangyue Zhang

**Affiliations:** 1 Institute of Stomatology, Nanjing Medical University, Nanjing, China; 2 Section of Clinical Epidemiology, Jiangsu Key Lab of Cancer Biomarkers, Prevention and Treatment, Cancer Center, Nanjing Medical University, Nanjing, China; 3 Department of Epidemiology and Biostatistics, MOE Key Laboratory of Modern Toxicology, School of Public Health, Nanjing Medical University, Nanjing, China; 4 Central Lab, Huai’an First People’s Hospital, Nanjing Medical University, Huai’an, China; MOE Key Laboratory of Environment and Health, Huazhong University of Science and Technology, China

## Abstract

**Backgrounds:**

The activation of Toll-like receptors (TLRs) may be an important event in the immune evasion of tumor cell. Recently, numerous studies have investigated the associations between *TLR2* −196 to −174 del and two SNPs of *TLR4* (rs4986790 and rs4986791) and the susceptibility to different types of cancer; however, the results remain conflicting. The aim of this study was to assess the association between *TLR2* and *TLR4* polymorphisms and cancer risk in a meta-analysis with eligible published studies.

**Methodology/Principle Findings:**

A dataset composed of 14627 cases and 17438 controls from 34 publications were included in a meta-analysis to evaluate the association between overall cancer risk or cancer-specific risk and three SNPs of *TLRs* (*TLR2* −196 to −174 del, *TLR4* rs4986790 and rs4986791). The results showed that all of these three polymorphisms were significantly associated with the increased cancer risk (dominant model: OR = 1.64, 95% CI: 1.04–2.60 for *TLR2* −196 to −174 del; OR = 1.19, 95% CI: 1.01–1.41 for *TLR4* rs4986790; and OR = 1.47, 95% CI: 1.120–1.80 for *TLR4* rs4986791; respectively). In stratified analysis, we found the effect of *TLR2* −196 to −174 del on cancer risk remained significant in the subgroup of Caucasians and South Asians, but not in East Asians. However, the association between rs4986791 and cancer risk was significant in both South Asians and East Asians, but not in Caucasians. Furthermore, the association between rs4986790 and cancer risk was statistically significant in digestive cancers (dominant model: OR = 1.76, 95% CI: 1.13–2.73) and female-specific cancers (dominant model: OR = 1.50, 95% CI: 1.16–1.94). However, no significant association with risk of digestive system cancers was observed for *TLR2* −196 to −174 del and *TLR4* rs4986791.

**Conclusions/Significance:**

This meta-analysis presented additional evidence for the association between *TLR2* and *TLR4* polymorphisms and cancer risk. Further well-designed investigations with large sample sizes are required to confirm this conclusion.

## Introduction

Toll-like receptors (TLRs) are a family of membrane-spanning innate immune receptors that recognize ligands derived from bacteria, fungi, viruses, and parasite [1]. TLRs play a key role in the realization of innate and adaptive immune response, being involved in the regulation of inflammatory reactions and activation of the adaptive immune response to eliminate infectious pathogens and cancer debris [2], [3]. Besides driving inflammatory responses, TLRs also regulate cell proliferation and survival by expanding useful immune cells and integrating inflammatory responses and tissue repair processes [4]. Furthermore, functional TLRs are expressed not only in immune cells, but also in cancer cells, thus implicating a role of TLRs in tumor biology [5], [6]. Increasing bodies of evidence have suggested that TLRs can act as a double-edged sword in cancer cells because uncontrolled TLR signaling provides a microenvironment that is necessary for tumor cells to proliferate and evade the immune response [4], [7]. In addition, activation of TLRs not only leads to the up-regulation of cellular defense mechanisms, but also results in up-regulation of DNA repair genes and increased functional DNA repair [8], [9].

The TLR family includes 2 subgroups, extracellular and intracellular, depending on their cellular localization. TLR1, 2, 5, 6 and 10 are extracellular TLRs, which are largely localized on the cell surface. Conversely, TLR3, 7, 8 and 9 (intracellular TLRs) are localized in intracellular organelles. The subcellular localization of *TLR4* is unique because it is localized to both the plasma membrane and endosomal vesicles [10]. *TLR2* and *TLR4* are major TLRs and have been actively investigated in inflammation and cancer. There is evidence that *TLRs*, particularly *TLR2* and *TLR4*, directly regulate major proinflammatory and host defense functions of human neutrophils [11]. Additionally, *TLR2* recognizes microbial pathogen-associated molecular patterns, such as cell wall peptidoglycan and lipoteichoic acid [12]. Positive *TLR2* expression in the tumor microenvironment suggests that immune surveillance is activated against the altered epithelial cells, whereas *TLR2* expression by malignant keratinocytes may be indicative of resistance to apoptosis as a prosurvival mechanism [13]. *TLR4* ligation on tumor cells can enhance the secretion of immunosuppressive cytokines and induce resistance to TNF-related apoptosis-inducing ligand (TRAIL)-induced apoptosis [14], [15]. Studies have shown that lipopolysaccharide (LPS) ligation to *TLR4* promotes tumor cell adhesion and invasion in a murine model by acting NF-kappa B [16], and the silencing of *TLR4* increases tumor progression and metastasis in a murine model of breast cancer [17].

Genetic studies have identified a polymorphism of *TLR2* that causes a 22-bp nucleotide deletion (−196 to −174 del) in the promoter region, which may influence the promoter activity of *TLR2* and lead to the decreased transcription of *TLR2* gene. Additionally, two SNPs in *TLR4* have also been identified; one is an A→G substitution at 896 base pair (bp) which results in an aspartic acid to glycine replacement at the codon 299 (D299G, rs4986790) and the other is a C→T substitution at 1196 bp which results in a threonine to isoleucine exchange at codon 399 (T399I, rs4986791). It has been shown that these two polymorphisms (rs4986790 and rs4986791) affect the extracellular domain of the receptor and may cause decreased ligand recognition [18]. The associations of these three polymorphisms with cancer risk have been widely studied, including bladder cancer [19], [20], breast cancer [21], [22], gastric cancer [23]–[31], prostate cancer [32]–[37], hepatocellular cancer [38], [39], gallbladder cancer [40], cervical cancer [41], nasopharyngeal cancer [42], leukemia [43], melanoma [44], endometrial cancer [45], lymphoma [46]–[50], esophageal cancer [31] and colorectal cancer [51], [52]. However, the results remained inconsistent rather than conclusive.

Considering the relatively small sample size in each single study might have low power to detect the effect of the polymorphisms on cancer risk and the underlying heterogeneity among different studies need be explored, we conducted a meta-analysis on all eligible published case-control studies to establish a relatively comprehensive picture of the relationship between these genetic variants (−196 to −174 del in *TLR2*, rs4986790 and rs4986791 in *TLR4*) and cancer risk.

## Materials and Methods

### Selection Criteria and Identification of Eligible Studies

Candidate studies were identified through computer-aided literature searching in PubMed for relevant articles in English and Chinese (last search was in January, 2013). The following keywords were used for this search: ‘*TLR2* or Toll like receptor 2′ or ‘*TLR4* or Toll like receptor 4′ and ‘cancer’ and ‘polymorphism’. We also included additional studies by a hands-on search of references of original studies. Abstracts, case-only articles, editorials, review articles and repeated literatures were excluded. The inclusion criteria of studies in the current meta-analysis were defined as follows: (1) original papers containing independent data; (2) case-control design on the association of *TLR2* (−196 to −174 del) or *TLR4* (rs4986790 and rs4986791) polymorphisms and cancer risk; (3) providing sufficient information to calculate the odds ratio (OR) or *P*-value; (4) written in English or Chinese.

### Data Extraction

Two investigators (Zhu LB and Jiang T) independently extracted data and reached a consensus on all items. For each study, the following information was extracted: first author, publication date, country, ethnicity, total number of cases and controls, the numbers of cases and controls grouped by different genotypes and Hardy-Weinberg equilibrium test in control subjects.

### Statistical Analysis

The crude odds ratios (ORs) and 95% confidence intervals (95% CIs) of *TLR2* (−196 to −174 del) and *TLR4* (rs4986790 and rs4986791) polymorphisms and cancer risk were estimated for each study. In addition, we also performed stratification analyses by cancer types and races. Digestive system included gastric, esophageal, colorectal, gallbladder and hepatocellular cancer; blood system included leukemia and lymphoma; female-specific included endometrial, breast and cervical cancer; male-specific included prostate cancer. If one cancer type contained less than three individual studies, they were combined into the ‘other’ group. All subjects were categorized as Caucasian, East Asian (China and Japan), South Asian (India) and mixed. The pooled ORs were performed by allele comparisons and genetic models comparisons. The HWE was assessed via χ^2^ test. A Chi-square based Q test and *I^2^*-statistic test were performed to assess the potential heterogeneity among the studies [53]. If the result of heterogeneity test was *p*>0.05, ORs were pooled according to the fixed-effect model [54]. Otherwise, the random-effect model was used [55]. The significance of the pooled ORs was determined by the Z-test. The sensitivity analysis was carried out to test the stability of the pooled effect by excluding each study individually and recalculating the ORs and 95% CI. To further explore the potential sources of heterogeneity among studies, meta regression was performed with some study characteristics, including ethnicity, genotyping methods, tumor types, sample size(≥500 or <500), minor allele frequency (MAF) in control subjects, and source of controls (population-based or hospital-based). Additionally, the inverted funnel plots and Begg’s funnel plot were used to evaluate publication bias [56]. The statistical analyses were performed by STATA 12.0 software. All *P* values were two-sided.

## Results

### Characteristics of Studies

**Figure 1:**
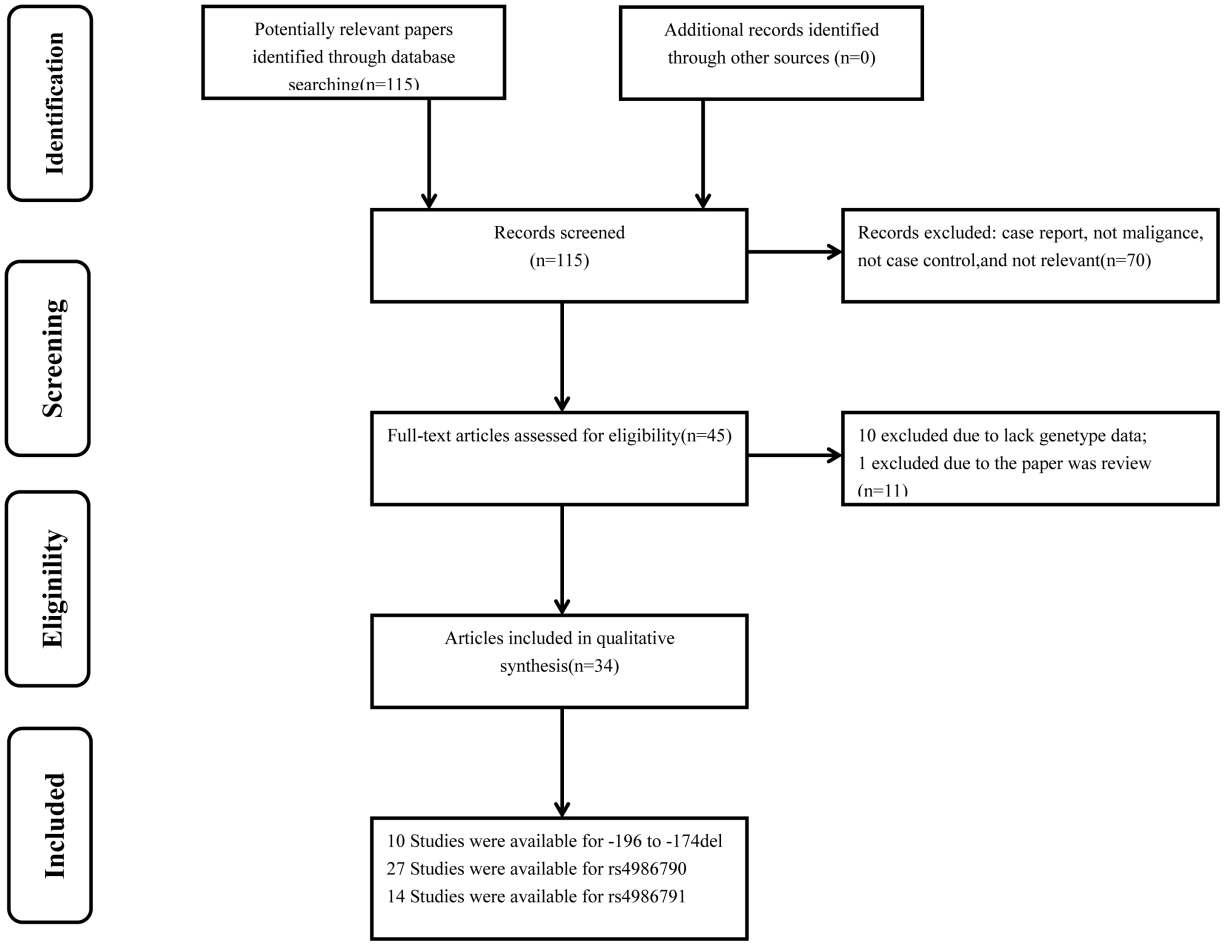
Study selection process.

115 articles were initially identified. Among them, 70 papers did not meet our criteria and were excluded. After reading the full texts of the remaining 45 papers, we found 10 papers had not enough genotype data and 1 paper was a review. Therefore, a total of 34 publications including 51 studies were remained (Figure 1). All studies were of case-control design, including fourteen kinds of cancers. Among them, 10 case-control studies focused on *TLR2* −196 to −174 del (2521 cases and 3247 controls), 27 on *TLR4* rs4986790 (9743 cases and 10839 controls), and 14 on *TLR4* rs4986791 (2363 cases and 3352 controls). Moreover, three publications focused on all three SNPs, ten publications focused on two SNPs, and twenty-one publications focused on only one SNP of all. The detailed characteristics of these studies, including first author, year of publication, country, ethnicity, cancer type, numbers of cases and controls, minor allele frequency (MAF) and HWE for all studies were summarized in Table 1. The distribution of genotypes in the controls of the studies was all in agreement with HWE except for four studies [20], [35], [40], [42].

**Table 1:** Characteristics of literatures included in the meta-analysis.

Reference	Year	Country	Ethnicity	Cancer type	Genotype-case			Genotype-control			MAF	HWE
***TLR2*** −**196 to** −**174 del**					ins/ins	ins/del	del/del	ins/ins	ins/del	del/del		
**Singh V^17^**	2012	India	South Asian	Bladder	110	79	11	119	73	8	0.223	0.437
**Theodoropoulos GE^19^**	2012	Greece	Caucasian	Breast	120	113	28	432	46	2	0.052	0.518
**de Oliveira JG^21^**	2012	Brazil	Caucasian	Gastric	116	50	8	189	34	2	0.084	0.733
**Mandal RK^30^**	2012	India	South Asian	Prostate	135	54	6	193	52	5	0.124	0.500
**Zeng HM^22^**	2011	China	East Asian	Gastric	119	110	19	187	246	63	0.375	0.195
**Nischalke HD^36^**	2011	Germany	Caucasian	Hepatocellular	115	63	11	248	92	7	0.153	0.649
**Hishida A^23^**	2010	Japan	East Asian	Gastric	243	267	73	304	316	79	0.339	0.819
**Srivastava K^38^**	2010	India	South Asian	Gallbladder	132	94	6	163	87	4	0.187	**0.044**
**Pandey S^39^**	2009	India	South Asian	Cervical	102	43	5	114	35	1	0.123	0.333
**Tahara T^28^**	2007	Japan	East Asian	Gastric	126	112	51	73	65	8	0.277	0.182
***TLR4*** ** rs4986790**					AA	AG	GG	AA	AG	GG		
**Theodoropoulos GE^19^**	2012	Greece	Caucasian	Breast	201	57	3	412	63	5	0.076	0.148
**de Oliveira JG^21^**	2012	Brazil	Caucasian	Gastric	154	20	0	215	10	0	0.022	0.773
**Yang ZH^40^**	2012	China	East Asian	Nasopharyngeal	205	29	2	250	33	4	0.071	**0.024**
**Shen Y^18^**	2012	China	East Asian	Bladder	431	2	3	519	1	2	0.005	**0.000**
**Miedema KG^41^**	2011	Netherlands	Caucasian	Leukemia	168	20	0	151	28	0	0.078	0.256
**Gast A^42^**	2011	Germany	Caucasian	Malignant Melanoma	665	91	0	659	73	3	0.054	0.525
**Ashton KA^43^**	2010	Australia	Caucasian	Endometrial	163	25	3	258	31	2	0.060	0.326
**Balistreri CR^31^**	2010	Italy	Caucasian	Prostate	49	1	0	111	13	1	0.060	0.383
**Rigoli L^27^**	2010	Italy	Caucasian	Gastric	42	18	0	80	7	0	0.023	0.696
**Etokebe GE^20^**	2009	Croatia	Caucasian	Breast	110	20	0	84	15	0	0.076	0.449
**Pandey S^39^**	2009	India	South Asian	Cervical	114	35	1	123	26	1	0.093	0.767
**Purdue MP^44^**	2009	US	Mixed	Non-Hodgkin lymphoma	1195	133	6	1126	131	8	0.058	0.055
**Wang MH^32^**	2009	US	Caucasian	Prostate	230	24	0	216	35	0	0.070	0.235
**Trejo-de la OA^24^**	2008	Mexico	Mixed	Gastric	34	4	0	138	6	0	0.021	0.798
**Ture-Ozdemir F^46^**	2008	Greece	Caucasian	Gastric MALT lymphoma	38	18	0	39	12	0	0.118	0.341
**Santini D^25^**	2008	Italy	Caucasian	Gastric	159	11	1	140	11	0	0.036	0.642
**Garza-Gonzalez E^26^**	2007	Mexico	Mixed	Gastric	72	6	0	175	14	0	0.037	0.518
**Hold GL^29^**	2007	Poland, US	Caucasian	Gastric	414	79	3	451	47	2	0.041	0.518
**Hold GL^29^**	2007	US	Mixed	Oesophageal	97	10	0	194	16	1	0.043	0.299
**Cheng I^35^**	2007	US	Mixed	Prostate	439	66	1	456	48	2	0.051	0.544
**Nieters A^45^**	2006	Germany	Caucasian	Lymphoma	590	84	1	596	71	1	0.055	0.456
**Boraska Jelavic T^49^**	2006	Croatia	Caucasian	Colorectal	77	10	2	84	4	0	0.023	0.827
**Landi S^50^**	2006	Spain	Caucasian	Colorectal	251	31	0	232	37	0	0.069	0.226
**Forrest MS^47^**	2006	US/UK	Caucasian	Non-hodgkin lymphoma	794	106	3	1254	172	6	0.064	0.969
**Hellmig S^48^**	2005	Germany/Austria	Caucasian	Gastric MALT lymphoma	83	4	0	313	45	0	0.063	0.204
**Chen YC^33^**	2005	USA	Caucasian	Prostate	588	66	3	605	59	5	0.052	**0.011**
**Zheng SL^34^**	2004	Sweden	Caucasian	Prostate	1241	136	1	693	79	5	0.057	0.103
***TLR4*** ** rs4986791**					CC	CT	TT	CC	CT	TT		
**Singh V^17^**	2012	India	South Asian	Bladder	163	35	2	173	26	1	0.070	0.983
**Theodoropoulos GE^19^**	2012	Greece	Caucasian	Breast	253	8	0	466	14	0	0.015	0.746
**de Oliveira JG^21^**	2012	Brazil	Caucasian	Gastric	165	9	0	219	6	0	0.013	0.839
**Yang ZH^40^**	2012	China	East Asian	Nasopharyngeal	188	45	3	254	32	1	0.059	0.994
**Agundez JA^37^**	2012	Spain	Caucasian	Hepatocellular	143	12	0	341	47	2	0.065	0.783
**Shen Y^18^**	2012	China	East Asian	Bladder	433	1	2	517	3	2	0.007	**0.000**
**Srivastava K^38^**	2010	India	South Asian	Gallbladder	195	32	5	232	24	1	0.051	0.656
**Balistreri CR^31^**	2010	Italy	Caucasian	Prostate	48	2	0	118	7	0	0.028	0.747
**Rigoli L^27^**	2010	Italy	Caucasian	Gastric	57	13	0	81	6	0	0.034	0.739
**Pandey S^39^**	2009	India	South Asian	Cervical	127	21	2	133	16	1	0.060	0.505
**Trejo-de la OA^24^**	2008	Mexico	Mixed	Gastric	57	4	0	193	9	0	0.022	0.746
**Santini D^25^**	2008	Italy	Caucasian	Gastric	155	15	1	147	4	0	0.013	0.869
**Garza-Gonzalez E^26^**	2007	Mexico	Mixed	Gastric	77	1	0	179	10	0	0.026	0.709
**Boraska Jelavic T^49^**	2006	Croatia	Caucasian	Colorectal	77	12	0	82	5	0	0.029	0.783

### Meta-analysis Results

The main results of this meta-analysis were listed in Table 2 and Figure S1. For *TLR2* polymorphism (−196 to −174 del), the meta-analysis showed a significantly increased risk for all cancers (allele comparison: OR = 1.62, 95% CI: 1.09–2.43, *P*<0.001 for heterogeneity test; dominant model: OR = 1.64, 95% CI: 1.04–2.60, *P*<0.001 for heterogeneity test; recessive model: OR = 2.28, 95% CI: 1.23–4.20, *P*<0.001 for heterogeneity test). Similarly, both of *TLR4* rs4986790 (allele comparison: OR = 1.17, 95% CI: 1.00–1.37, *P*<0.001 for heterogeneity test; dominant model: OR = 1.19, 95% CI: 1.01–1.41, *P*<0.001 for heterogeneity test) and rs4986791 (allele comparison: OR = 1.47, 95% CI: 1.21–1.78, *P* = 0.070 for heterogeneity test; dominant model: OR = 1.47, 95% CI: 1.20–1.80, *P* = 0.078 for heterogeneity test) also significantly increased the overall cancer risk.

**Table 2:** Associations between *TLRs* polymorphisms and overall cancer risk by races.

Polymorphism	Ethnicities	Studies	Allele comparison		Dominant model		Recessive model	
			OR(95% CI)	*p* *	OR(95% CI)	*p* *	OR(95% CI)	*p* *
−**196 to** −**174 del**	Total	10	**1.62(1.09–2.43)**	<0.001	**1.64(1.04–2.60)**	<0.001	**2.28(1.23–4.20)**	<0.001
	Caucasian	3	**3.29(1.14–9.51)**	<0.001	**3.56(1.10–11.51)**	<0.001	**7.29(1.75–30.37)**	0.029
	East Asian	3	1.04(0.71–1.52)	<0.001	0.96(0.66–1.40)	<0.001	1.27(0.55–2.95)	<0.001
	South Asian	4	**1.32(1.11–1.58)**	0.785	**1.37(1.11–1.68)**	0.870	1.72(0.94–3.14)	0.751
**rs4986790**	Total	27	**1.17(1.00–1.37)**	<0.001	**1.19(1.01–1.41)**	<0.001	–	–
	Caucasian	19	1.17(0.95–1.45)	<0.001	1.18(0.95–1.47)	<0.001	–	–
	East Asian	2	1.04(0.79–1.36)	0.770	1.08(0.81–1.45)	0.797	–	–
	South Asian	1	1.37(0.82–2.30)	<0.001	1.44(0.82–2.52)	<0.001	–	–
	Mixed	5	1.05(0.87–1.27)	0.348	1.08(0.89–1.32)	0.320	–	–
**rs4986791**	Total	14	**1.47(1.21–1.78)**	0.070	**1.47(1.20–1.80)**	0.078	–	–
	Caucasian	7	1.51(0.84–2.71)	0.023	1.55(0.85–2.83)	0.023	–	–
	East Asian	2	**1.72(1.14–2.62)**	0.198	**1.77(1.12–2.77)**	0.192	–	–
	South Asian	3	**1.58(1.16–2.16)**	0.718	**1.55(1.11–2.17)**	0.846	–	–
	Mixed	2	0.75 (0.28–2.01)	0.117	0.75(0.28–2.02)	0.114	–	–

The results were in bold, if the *P*<0.05.

* *P* values for heterogeneity test. If the result of the heterogeneity test was *p*>0.05, ORs were pooled according to the fixed-effect model. Otherwise, the random-effect model was used.

We further performed stratification analysis by ethnicity and cancer types. The results indicated that variant genotypes of *TLR2* −196 to −174 del tended to be associated with overall cancer risk in Caucasians (allele comparison: OR = 3.29, 95% CI: 1.14–9.51, *P*<0.001 for heterogeneity test; dominant model: OR = 3.56, 95% CI: 1.10–11.51, *P*<0.001 for heterogeneity test) and South Asians (allele comparison: OR = 1.32, 95% CI: 1.11–1.58, *P* = 0.785 for heterogeneity test; dominant model: OR = 1.37, 95% CI: 1.11–1.68, *P* = 0.870 for heterogeneity test), but not in East Asians (Table 2). However, the association between rs4986791 and cancer risk was significant in both South Asians (allele comparison: OR = 1.58, 95% CI: 1.16–2.16, *P* = 0.718 for heterogeneity test; dominant model: OR = 1.55, 95% CI: 1.11–2.17, *P* = 0.846 for heterogeneity test) and East Asians (allele comparison: OR = 1.72, 95% CI: 1.14–2.62, *P* = 0.198 for heterogeneity test; dominant model: OR = 1.77, 95% CI: 1.12–2.77, *P* = 0.192 for heterogeneity test), but not in Caucasians (Table 2). When stratified by cancer types, significantly increased risks of *TLR4* rs4986790 were found in digestive cancers (allele comparison: OR = 1.79, 95% CI: 1.14–2.81, *P* = 0.001 for heterogeneity test; dominant model: OR = 1.76, 95% CI: 1.13–2.73, *P* = 0.003 for heterogeneity test) and female-specific cancers (allele comparison: OR = 1.44, 95% CI: 1.14–1.83, *P* = 0.641 for heterogeneity test; dominant model: OR = 1.50, 95% CI: 1.16–1.94, *P* = 0.537 for heterogeneity test), but not in blood cancers or male-specific cancers (Table 3). However, no significant association with risk of digestive cancers was observed for *TLR2* −196 to −174 del and *TLR4* rs4986791 (Table 3). We also further investigated the associations between three SNPs and gastric cancer or prostate cancer (involved in more than three studies) and found that both *TLR4* rs4986790 (allele comparison: OR = 2.18, 95% CI: 1.67–2.84, *P* = 0.068 for heterogeneity test; dominant model: OR = 2.20, 95% CI: 1.67–2.89, *P* = 0.104 for heterogeneity test) and rs4986791 (allele comparison: OR = 1.90, 95% CI: 1.20–3.12, *P* = 0.193 for heterogeneity test; dominant model: OR = 1.98, 95% CI: 1.22–3.21, *P* = 0.104 for heterogeneity test) were associated with a significantly increased risk of gastric cancer, but not *TLR2* −196 to −174 del (Table 4). Furthermore, we did not observe significant association between rs4986790 and prostate cancer risk.

**Table 3:** Associations between *TLRs* polymorphisms and overall cancer risk by cancer types.

Polymorphism	Cancer type	Studies	Allele comparison		Dominant model		Recessive model	
			OR(95% CI)	*p* *	OR(95% CI)	*p* *	OR(95% CI)	*p* *
−**196 to** −**174 del**	Digestive	6	1.32(0.97–1.79)	<0.001	1.29(0.92–1.80)	<0.001	1.74(0.91–3.34)	<0.001
	Others	4	2.19(0.82–5.82)	<0.001	1.32(0.80–6.77)	<0.001	3.93(0.89–17.47)	0.002
**rs4986790**	Digestive	9	**1.79(1.14–2.81)**	0.001	**1.76(1.13–2.73)**	0.003	–	–
	Blood	6	0.95(0.83–1.10)	0.170	0.95(0.83–1.11)	0.140	–	–
	Female-specific	4	**1.44(1.14–1.83)**	0.641	**1.50(1.16–1.94)**	0.537	–	–
	Male-specific	5	0.95(0.80–1.13)	0.068	0.99(0.82–1.18)	0.062	–	–
	other	3	1.11(0.87–1.43)	0.535	1.16(0.89–1.52)	0.666	–	–
**rs4986791**	Digestive	8	1.58(0.93–2.69)	0.014	1.60(0.94–2.74)	0.017	–	–
	Others	6	**1.47(1.13–1.92)**	0.607	**1.47(1.11–1.96)**	0.589	–	–

The results were in bold, if the *P*<0.05.

* *P* values for heterogeneity test. If the result of the heterogeneity test was *p*>0.05, ORs were pooled according to the fixed-effect model. Otherwise, the random-effect model was used.

**Table 4:** Main result of pooled odds ratios (ORs) in gastric and prostate cancer.

Polymorphism	Cancer type	Studies	Allele comparison		Dominant model		Recessive model	
			OR(95% CI)	*p* *	OR(95% CI)	*p* *	OR(95% CI)	*p* *
−**196 to** −**174 del**	Gastric cancer	4	1.27(0.83–1.95)	<0.001	1.21(0.75–1.94)	<0.001	1.58(0.70–3.59) <0.001	
**rs4986790**	Gastric cancer	6	**2.18(1.67–2.84)**	0.068	**2.20(1.67–2.89)**	0.104	–	–
	Prostate cancer	5	0.95(0.80–1.13)	0.068	0.99(0.82–1.18)	0.062	–	–
**rs4986791**	Gastric cancer	5	**1.93(1.20–3.12)**	0.193	**1.98(1.22–3.21)**	0.177	–	–

The results were in bold, if the *P*<0.05.

* *P* values for heterogeneity test. If the result of the heterogeneity test was *p*>0.05, ORs were pooled according to the fixed-effect model. Otherwise, the random-effect model was used.

### Test of Heterogeneity

A meta-regression was conducted to explore the possible source of heterogeneity for −196 to −174 del and rs4986790 because both of *P* values for heterogeneity test were less than 0.05 in the comparisons. We identified that MAFs of −196 to −174 del and rs4986790 were significant sources of heterogeneity (*P* = 0.008 for −196 to −174 del, *P* = 0.039 for rs4986790, respectively). We also found that ethnicity was a significant source of heterogeneity for −196 to −174 (*P* = 0.036). However, genotyping methods, tumor types, sample size, and source of controls could not substantially influence the initial heterogeneity.

### Sensitivity Analyses and Publication Bias

The leave-one-out sensitivity analysis indicated that no single study changed the pooled ORs qualitatively (data not shown). Furthermore, we also conducted a sensitivity analysis on the *TLR2* and *TLR4* polymorphism and risk of cancer by excluding all four studies departure from HWE among controls [20], [35], [40], [42] and their exclusion did not substantially affect the results of the meta-analysis (dominant model: OR = 1.68, 95% CI: 1.00–2.81 for −196 to −174del; dominant model: OR = 1.20, 95% CI: 1.00–1.44 for rs4986790; dominant model: OR = 1.49, 95% CI: 1.21–1.83 for rs4986791).

**Figure 2:**
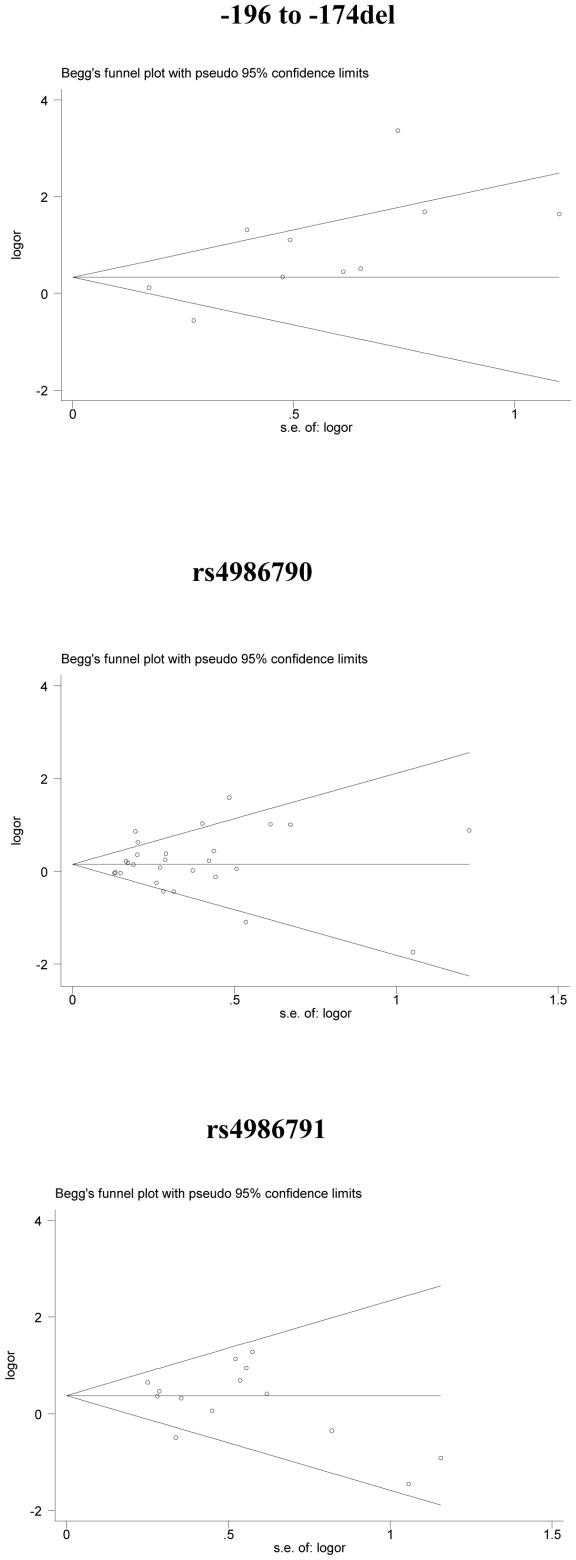
Begg’s funnel plot for publication bias test. Each point represents a separate study for the indicated association. s.e., standardized effect.

The inverted funnel plots (Figure 2) and Begg’s test were performed to assess the publication bias, and the results did not suggest any obvious evidence of asymmetry for *TLR2* and *TLR4* polymorphisms (*P* = 0.152 for −196 to −174 del; *P* = 0.505 for rs4986790; *P* = 0.324 for rs4986791, respectively).

## Discussion

In this meta-analysis of 34 independent publications, we found that three genetic variants of *TLRs* (*TLR2* −196 to −174 del, *TLR4* rs4986790 and rs4986791) were significantly associated with an increased risk of overall cancers. Furthermore, the stratification analysis showed that the risk effect of polymorphisms was more prominent in subjects with some special races or cancer types. All these findings suggested that polymorphisms of *TLR2* and *TLR4* might contribute to risk of human cancer.

The −196 to −174 del polymorphism in *TLR2* located on chromosome 4 causes a 22-bp nucleotide deletion and it has been recently proposed to reflect differential trans-activation of *TLR2* promoter constructs and expression levels of *TLR2* [38]. However, population studies showed that *TLR2* −196 to −174 del polymorphism might play conflicting roles for the risk of different types of cancer. For example, it was reported that the *TLR2* −196 to −174 del polymorphism was associated with risk of several cancers, such as cervical cancer, gastric cancer, breast cancer and hepatocellular cancer [21], [23], [24], [38], [41], but not associated with other cancers including bladder, prostate cancer and gallbladder cancer [19], [32], [40]. And even the same kind of cancer, the results were inconsistent [23], [25]. To comprehensively investigate the effect of this polymorphism on the risk of overall cancers, we conducted this meta-analysis and found that *TLR2* −196 to −174 del polymorphism significantly increased risk of cancers, supporting the hypothesis that this SNP plays a role in changed expression of *TLR2* and cancer development.

The *TLR4* gene is mapped on chromosome 9 and consists of three exons. In exon 3, two non-synonymous SNPs (+896A/G rs4986790 and +1196C/T rs4986791) induces the substitution of amino acids Asp299Gly and Thr399Ile, respectively. The substitution of Asp299Gly disrupts the normal structure of the extracellular region of the *TLR4*, which may cause decreased ligand recognition or protein interaction and decreased responsiveness to lipopolysaccharide [57]. Consequently, such change can affect the transport of *TLR4* to the cell membrane and lead to an exaggerated inflammatory response with severe tissue destruction. The results of previous studies regarding the association between these two SNPs and cancer risk were inconsistent. These pooled analysis did not find any significant association between the two SNPs and risk of prostate cancer [58] or gastric cancer [59]. However, a recent meta-analysis of 22 publications on six selected SNPs (rs1927914, rs4986790, rs4986791, rs11536889, rs1927911 and rs2149356) in *TLR4* and cancer risk reported that *TLR4* rs4986790 and rs4986791 were significantly associated with increased risk of overall cancer and significantly elevated risk of gastric cancer was observed for rs4986790 in a stratification study [60]. Our meta-analysis including more studies (27 studies for rs4986790 and 14 studies for rs4986791) and more cancer types provided additional evidence that these two SNPs may play a role in the development of cancer. In the stratification analysis by cancer types, we found that the effect of rs4986790 on cancer risk was more evident in female-specific cancers and digestive cancers, especially for gastric cancer. Similarly, the risk effect of rs4986791 was also prominent in gastric cancer. Studies have shown that H.pylori activates *TLR4* expression in gastric epithelial cells and TLR4 can serve as a receptor for H.pylori binding [61], [62]. Thus, potentially functional polymorphisms of *TLR4* may affect the function of *TLR4* and contribute to H. pylori-associated carcinogenesis. An important reason for the different findings by previously performed studies may be the insufficient study power to detect modest effects of polymorphisms.

In term of stratified analyses by races, our findings indicated that *TLR2* −196 to −174 del had an significant association with cancer risk in Caucasians and South Asians, but not in East Asians. However, the association between *TLR4* rs4986791 and cancer risk was significant in both South Asians and East Asians, but not in Caucasians. These differences may be induced by different genetic backgrounds and environmental exposures, as indicated by the difference of minor allele frequency in controls among the two populations (Table 1). For example, the MAF of *TLR2* −196 to −174 del in Caucasian controls varied from 0.05 to 0.15, but that in Asians was from 0.12 to 0.38. Allele frequency might reflect the natural selection pressures or a balance by other related functional genetic variants and/or environmental exposures. We also searched some public databases, such as Hapmap (http://hapmap.ncbi.nlm.nih.gov/) and SNPinfo (http://snpinfo.niehs.nih.gov/), and found that rs4986790 was in high linkage disequilibrium (LD) with rs4986791 in Caucasians (r^2^ = 1), but not data was available in Asians because of low allele frequency of these two SNPs. In our analysis, the associations of rs4986790 and rs4986791 with cancer risk were consistent in Caucasians, but inconsistent in Asians. These findings further indicate that the effect of genetic variants on cancer risk may be different between multiple ethnic groups. Some limitations and potential bias should be addressed. First, the subgroups may have a relatively lower power based on a small number of studies. Second, a more precise analysis should be conducted, if individual data were available, allowing for the adjustment by some co-variants such as age, gender and other environmental factors. However, these information were unavailable from most of studies. Third, the controls in the included studies were recruited from different ways and not uniformly defined, which may have induced some bias for the meta-analysis. Last, the gene-gene interaction is important for the development of complex diseases including cancer because single genetic variation may only have a modest effect [63], [64]. However, the original genotyping data of each publication was unavailable and we could not carry out gene-gene interaction analysis in this study.

In conclusion, this meta-analysis provided statistical evidence that the *TLR2* and *TLR4* polymorphisms were associated with cancer risk, particularly for gastric cancer. However, due to the limitations of original studies included in the meta-analyses, well-designed prospective studies with larger samples are needed to confirm these findings.

## Supporting Information

Checklist S1 (DOC)

Figure S1 (DOC)
