# Supplementary material for: Association of TLR2 and TLR4 Polymorphisms with Risk of Cancer: A Meta-Analysis
Source: PLoS One. 2013 Dec 20;8(12):e82858. doi: 10.1371/journal.pone.0082858 (PMC3869723; doi:10.1371/journal.pone.0082858)
Supplement: Figure S1 — (DOC) [file pone.0082858.s002.doc]

**-196 to -174 del (allele) -196 to -174 del (dominant)**

**rs4986790 (allele) rs4986790 (dominant)**

**rs4986791 (allele) rs4986791 (dominant)**

**Supplemental Figure 1. Forest plots for the association between *TLR2* and *TLR4* polymorphisms and cancer risk.**
